# Histone Deacetylase Inhibition and Autophagy Modulation Induces a Synergistic Antiproliferative Effect and Cell Death in Cholangiocarcinoma Cells

**DOI:** 10.1021/acsomega.3c01317

**Published:** 2023-06-08

**Authors:** Münevver Yenigül, Emel Başak Gencer Akçok

**Affiliations:** † Graduate School of Engineering and Science, Bioengineering Department, Abdullah Gul University, Kayseri 38080, Turkey; ‡ Faculty of Life and Natural Sciences, Molecular Biology and Genetics Department, Abdullah Gul University, Kayseri 38080, Turkey

## Abstract

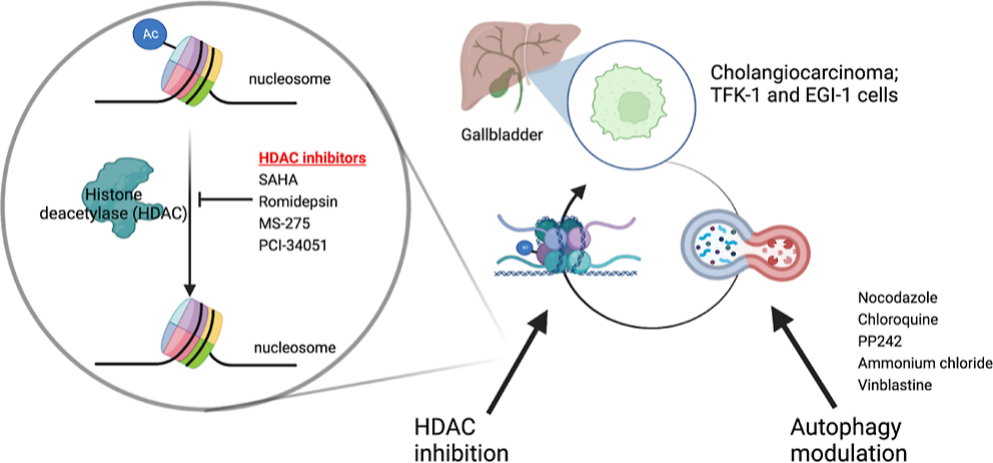

Cholangiocarcinoma, also known as biliary tract cancer, is an aggressive adenocarcinoma arising from epithelial cells lining the intra- and extrahepatic biliary system. The effects of autophagy modulators and histone deacetylase (HDAC) inhibitors in cholangiocarcinoma are not fully known. It is essential to understand the molecular mechanisms and the effects of HDAC inhibitors in the context of cholangiocarcinoma. The antiproliferative effect of different HDAC inhibitors and autophagy modulation was investigated by the MTT cell viability assay in TFK-1 and EGI-1 cholangiocarcinoma cell lines. Combination indexes were calculated using CompuSyn software. Consequently, apoptosis was detected by Annexin V/PI staining. The effect of the drugs on the cell cycle was measured by the propidium iodide staining. The HDAC inhibition was confirmed via acetylated histone protein levels by western blotting. HDAC inhibitors, MS-275 and romidepsin, showed a better synergistic effect with the nocodazole combination. The combination treatment exerted its growth inhibitory effect by cell cycle arrest and induction of apoptosis. The cell cycle analysis of the combination treatment showed that the S phase and G2/M phase were achieved. Moreover, the necrotic and apoptotic cell population increased after single HDAC inhibitors and combination treatment. The anti-cancer effect of HDAC inhibitors is revealed by acetylation levels of histones. While acetylation levels were increased in response to HDAC inhibitors and autophagy modulator combinations, the HDAC expression decreased. This study highlights the importance of the combination of HDAC inhibition and autophagy modulators and demonstrates a synergistic effect, which could be a promising therapy and novel treatment approach for cholangiocarcinoma.

## Introduction

Cholangiocarcinoma (CCA), also known as biliary tract cancer, is a heterogeneous group of malignancies formed by the differentiation of epithelial cells in the biliary tract.^1^ CCA is the second most common primary liver tumor and it has both an increasing rate and high mortality worldwide due to its late diagnosis, refractory type, and aggressiveness.^2^ The bile ducts are divided into intrahepatic or extrahepatic.^3^ Intrahepatic cholangiocarcinoma (iCCA) is the second most common primary tumor and accounts for approximately 10% of all CCAs.^4^ Unfortunately, treatment options for CCA are discouraging; therefore, novel therapeutic strategies should be developed against CCA. Recently, histone deacetylase inhibitors (HDACis) are presented as attractive anticancer agents. However, their mode of action in CCA is still poorly understood. Therefore, understanding the molecular mechanisms of HDAC inhibition in the context of CCA can provide insights into the development of this aggressive disease, where new therapeutic options are highly required.

The acetylation and deacetylation of histones recreate their critical role in tumorigenesis and cancer progression. Histone acetylation is regulated by histone acetyltransferase (HAT) and histone deacetylase (HDAC) enzymes. HATs remove an acetyl group from acetyl coenzyme A and transfer the acetyl group to lysine residues of histones with covalent bonds, hence causing the relaxation of the chromatin structure and the chromatin becomes transcriptionally active. On the other hand, HDAC enzymes remove acetyl groups from histone proteins.^5,6^ Histone deacetylation makes the chromatin structure more condensed and causes the suppression of gene expression. In some studies, it has been proven that HDAC enzymes play an active role in many cancer types, such as gastrointestinal,^7^ breast,^8,9^ lymphoblastic and myeloid leukemia,^10,11^ pancreas,^12^ and lung cancer.^13^ Histone deacetylases are highly expressed in both normal cholangiocytes and cholangiocarcinoma.^14^ Although the effects of HDACis are studied in CCA, not much is known about their mechanism.

Autophagy is described as a mechanism for cell survival, generally controlling and balancing the destruction, synthesis, and recycling of substances within the cell.^15^ Anticancer drugs known as autophagy modulators work by inhibiting or activating autophagy pathways. While PP242 is an autophagy activator for mTOR inhibitors, autophagy inhibition is achieved by using ammonium chloride and chloroquine for autolysosomal degradation, nocodazole and vinblastine for autophagosome-lysosome fusion.^16−18^ Autophagy has a complex role in cancer development. According to most studies, a decreased activity of some HDAC enzyme classes in cancer cells is considered a link to higher expression of autophagy regulators involved in the various cell functions. As such, simultaneous targeting autophagy with HDACis may improve the therapeutic effects against cancer.

Depending on the tissue context and the experimental setup, different studies have indicated HDACis to be inhibitors or activators of autophagy. Stankov et al. have shown that HDACis induce apoptosis in myeloid leukemia by suppressing autophagy.^11^ In addition to that, there has been a synergistic effect when HDACis are combined with autophagy inhibitors against prostate cancer, malignant glioma cells, and malignant sheath tumors.^19−21^ Thus, further studies are needed to decipher the efficacy of HDACis in different cancers.

Because the effect of autophagy modulators and HDAC inhibitors in CCA is not clearly known, this study focused on the effects of the combination of autophagy modulators with the inhibition of HDAC on CCA cells. The results of this study demonstrated an increased synergistic antiproliferation effect of the combination of HDAC inhibitors, SAHA, MS-275, and romidepsin with autophagy modulator, nocodazole, on the TFK-1 and EGI-1 cells. This research constitutes a new approach to the combination treatment of CCA.

## Experimental Procedures

### Chemicals

HDAC enzyme inhibitors; SAHA (Sigma), romidepsin (Selleckchem), MS-275 (Sigma), and PCl-34051 (Cayman chemical) were dissolved in DMSO (dimethylsulfoxide), autophagy modulators; nocodazole (Sigma), ammonium chloride (Millipore), and PP242 (Sigma) were dissolved in DMSO, chloroquine (Chemcruz) and vinblastine (Sigma) was dissolved in water as recommended by the supplier. The main stock solutions were prepared and stored at −20 °C. The RPMI-1640, FBS, penicillin/streptomycin, and PBS were purchased from Sigma, Biological Industries, Sigma, and Gibco, respectively.

### Cell Lines and Maintenance

EGI-1, TFK-1, and HepG2 cell lines were obtained from the German National Resource Center for Biological Material (DSMZ). They were cultured under the recommended conditions. All cell lines were cultured in the RPMI medium supplemented with 10% FBS and 100 U/mL penicillin/streptomycin at 37 °C in a 5% CO_2_ incubator.

### Cell Viability Assay

The viability of cells was assessed by the 3-(4,5-dimethylthiazol-2-yl)-2,5-diphenyl-2*H*-tetrazolium bromide (MTT) cell viability assay. All cell lines were seeded in triplicates in 96-well plates as 10,000 cells/100 μL per well. After overnight incubation, the cells were treated with DMSO as the control, HDAC inhibitors alone, autophagy modulators alone, and combinations of these for 48 h. After the incubation period, 10 μL of MTT solution was added to each well and the cells were incubated between 2 to 4 h at 37 °C in a 5% CO_2_ incubator. The plates were centrifuged at 1800 rpm for 10 min. The formed formazan crystals were solubilized with 100 μL of DMSO. Then, the plates were incubated for 15 min on the shaker and the absorbance was measured with a Varioskan LUX multimode microplate reader (Thermo Scientific) at 570 nm.

### Calculation of Combination Index

Combination analysis (isobologram analysis) was performed by using the Calcusyn 2.0 program (CompuSyn software, Biosoft, Cambridge, United Kingdom). Ccombination index (CI) values were calculated by the program. The effects of the drug combination that was used in this study were evaluated using the CI based on a Chou-Talalay’s multidrug effect equation. A CI of <1, =1, or >1 is indicative of synergistic, additive, or antagonistic effects, respectively.^22^

### Analysis of Cell Cycle Distribution

TFK-1 and EGI-1 cells were seeded as 1 × 10^6^ cells/well in a 6-well plate and incubated overnight. After, the cells were treated with inhibitors for 48 h alone or in combination. Then, the cells were harvested by trypsin and centrifuged at 260*g* for 10 min at 4 °C. The supernatant was removed and the pellet was washed with 1 mL cold PBS and then centrifuged at 260*g* for 10 min. The cells were resuspended with 1 mL cold PBS and then, 4 mL ethanol (70%) was added to each sample. The samples were homogenized gently via vortex. The samples were incubated for at least 24 h at −20°C for the fixation of the cells. Later, samples were centrifuged, and the supernatant was discarded. The cell pellet was resuspended in 5 mL cold PBS and then centrifuged. Then, PBS/Triton X-100 was added, followed by the addition of 100 μL of RNase A (Sigma), and incubated at 37 °C for 30 min. Finally, 100 μL of propidium iodide (Biolegend) was added and left at room temperature for 10 min. Cell cycle analysis was performed by flow cytometry (BD FACSAria III Cell Analyzer).

### Flow Cytometric Detection of Apoptosis by Annexin-V FITC/Propidium Iodide Dual Staining

Apoptotic cell death was assessed using Annexin V/FITC apoptosis detection kit as previously.^23^ Briefly, 1 × 10^6^ cells/well were treated with HDACis and nocodazole alone, and the IC30 combinations of both for 48 h in a 6-well plate. After incubation, the cells were collected at 1700 rpm for 5 min at 4 °C, washed with cold 1X PBS twice, and resuspended with 200 μL 1× annexin binding buffer. Then, 2 μL of annexin-V FITC (Biolegend) and 4 μL of propidium iodide were added to each obtained cell suspension. Following the incubation at room temperature for 15 min, apoptotic cells were detected using a flow cytometer (BD FACSAria III Cell Analyzer).

### Western Blot

1 × 10^6^ cells were treated with HDACis, nocodazole alone, and in combination for 48 h. The expression levels of HDAC1/2, acetylated histone 3 (Ac-H3), total histone 3 (H3), and acetylated histone 4 (Ac-H4) were checked by the western blot. Cells were lysed in RIPA buffer (50 mM Tris–HCl pH:8.0, 150 mM NaCl, 1% NP-40, 0.5% sodium deoxycholate, and 0.1% SDS, protease, and phosphatase inhibitors). The supernatants were collected and the concentration of protein was measured using RC DC protein assay kit (Bio-Rad, USA). 20 μg/well total protein was separated by 15% SDS-PAGE and transferred to PVDF membranes. The membranes were blotted with primary antibodies for HDAC1 (1:1000, Santa Cruz), HDAC2 (1:250, Santa Cruz), Ac-H3 (1:1000, Cell Signaling, USA), H3 (1:1000, Cell Signaling, USA), Ac-H4 (1:1000, Cell Signaling, USA), GAPDH (1:2000, Proteintech) overnight at 4 °C and conjugated with appropriate secondary antibodies [peroxidase affiniPure goat anti-rabbit IgG (1:10,000) peroxidase affiniPure goat anti-mouse IgG (1:10,000)]. The membranes were visualized with a Pierce ECL western blotting substrate kit (Thermo Scientific, USA). Immunoreactive bands and their densitometric analysis were carried out using imaging software (Bio-Rad, ChemiDoc, Image Lab, 3.0).

### Statistical Analysis

The data were analyzed by using GraphPad software (8.0.2, San Diego, CA). All results were expressed as the mean ± standard deviation (SD) from three independent experiments. Comparisons among three groups were evaluated using one-way ANOVA and two-way ANOVA by the Dunnett’s test. A value of *p* < 0.05 was considered to be statistically significant and a value of *p* < 0.0001 was considered to be a highly statistically significant difference.

## Results

### HDACis Treatment Effectively Inhibits the Proliferation of CCA and HCC Cells

**Figure 1:**
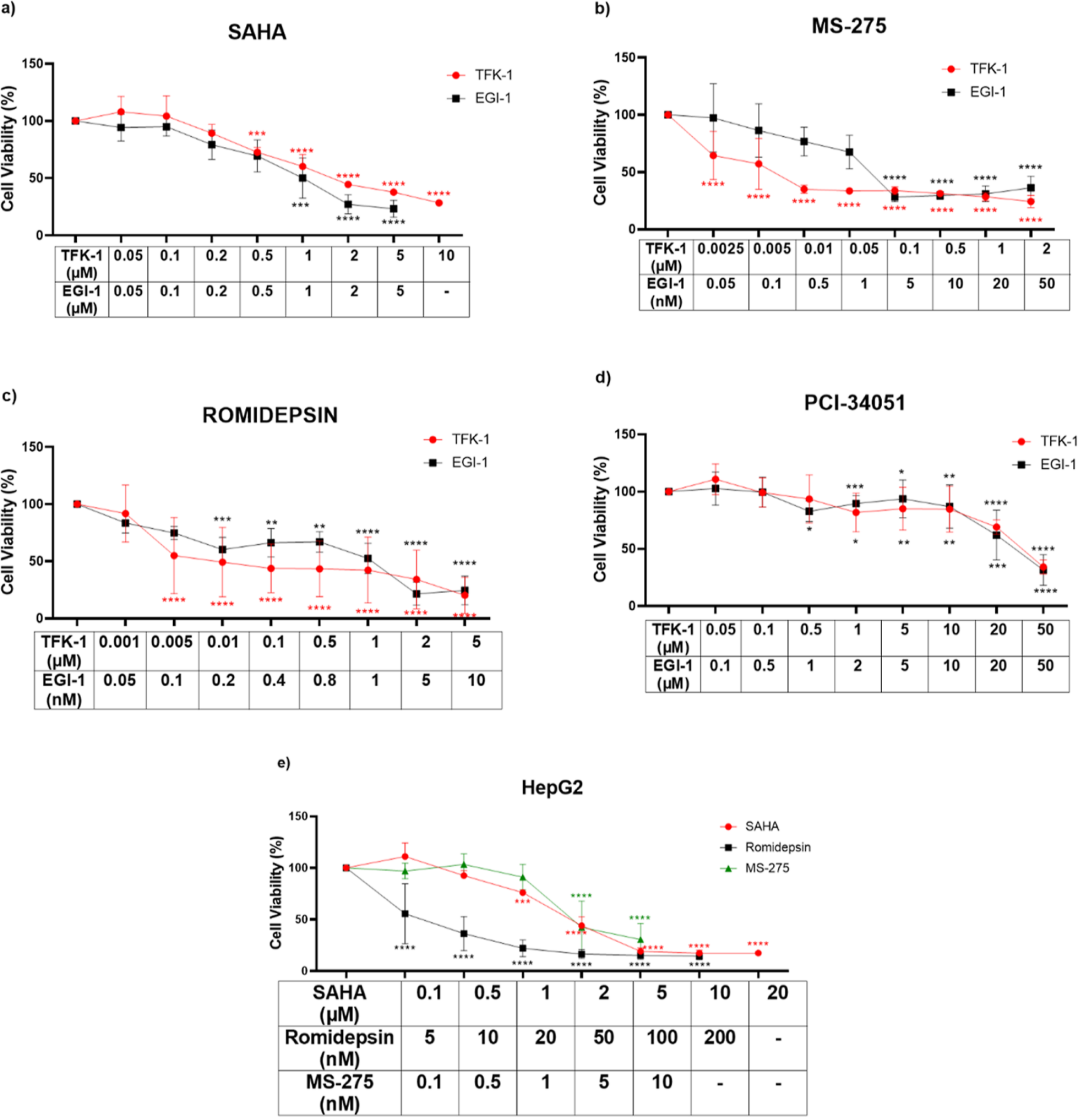
Cytotoxic effect of HDACis on TFK-1, EGI-1, and HepG2 cells. (a) SAHA, (b) MS-275, (c) romidepsin, and (d) PCI-34051 treatment on proliferation of TFK-1 and EGI-1 cells, (e) SAHA, romidepsin, and MS-275 treatment on the proliferation of HepG2 cells. Each set of experiments was averaged and statistical analysis was performed using two-way ANOVA by the Dunnett’s test. These results represent data from samples in triplicate across three independent experiments (*n* = 3). *P < 0.05, **P < 0.01, ***P < 0.001, and ****P < 0.0001.

The romidepsin, MS-275, PCI-34051, and SAHA, are inhibitors that target different HDAC enzyme classes. First, we tested this panel of HDAC inhibitors on CCA cell lines and determined the growth inhibitory effects of these HDACis by the MTT assay. Treating EGI-1 and TFK-1 cells with HDACis reduced the growth of both cell lines in a dose-dependent manner compared to the DMSO control (Figure 1). As presented in Figure 1a–c, the CCA cells treated with SAHA, MS-275, and romidepsin were greatly effective at low doses compared to PCI-34051 (Figure 1d). IC30 values of HDACis were calculated for TFK-1 and EGI-1 cells (Table 1). The most effective inhibitors selected from this panel were MS-275, SAHA, and romidepsin. These inhibitors were administered to the colon cancer cell line, HepG2, which we used as a control (Figure 1e). IC30 values of MS-275, SAHA, and romidepsin were calculated for HepG2 cells, as well (Table 1). Our results demonstrated that romidepsin, MS-275, and SAHA inhibitors showed a better anti-proliferative effect on both cell lines at low doses compared to PCI-34051, so these three inhibitors were used in further experiments.

**Table 1:** IC30 values of Selected HDACis and Autophagy Modulators on TFK-1, EGI-1, and HepG2 Cells^a^

cell lines		TFK-1	EGI-1	HepG2
HDACis	MS-275	3.5 nM ± 0.31	0.53 nM ± 0.21	4.3 nM ± 0.67
	SAHA	2.25 μM ± 0.12	0.43 μM ± 0.18	1.2 μM ± 0.08
	romidepsin	3.7 nM ± 0.56	0.74 nM ± 0.26	0.94 nM ± 0.16
autophagy modulators	chloroquine	3.94 μM ± 0.4	5.14 μM ± 0.25	4.1 μM ± 0.08
	nocodazole	2.89 μM ± 0.39	2.15 μM ± 0.54	4.7 μM ± 0.16
	PP242	1.1 nM ± 0.23	9.02 nM ± nd	4.4 μM ± 0.17

a Values are expressed as mean ± SD. n.d.—not defined.

### Autophagy Modulators Inhibited Cell Proliferation of CCA and HCC Cell Lines

**Figure 2:**
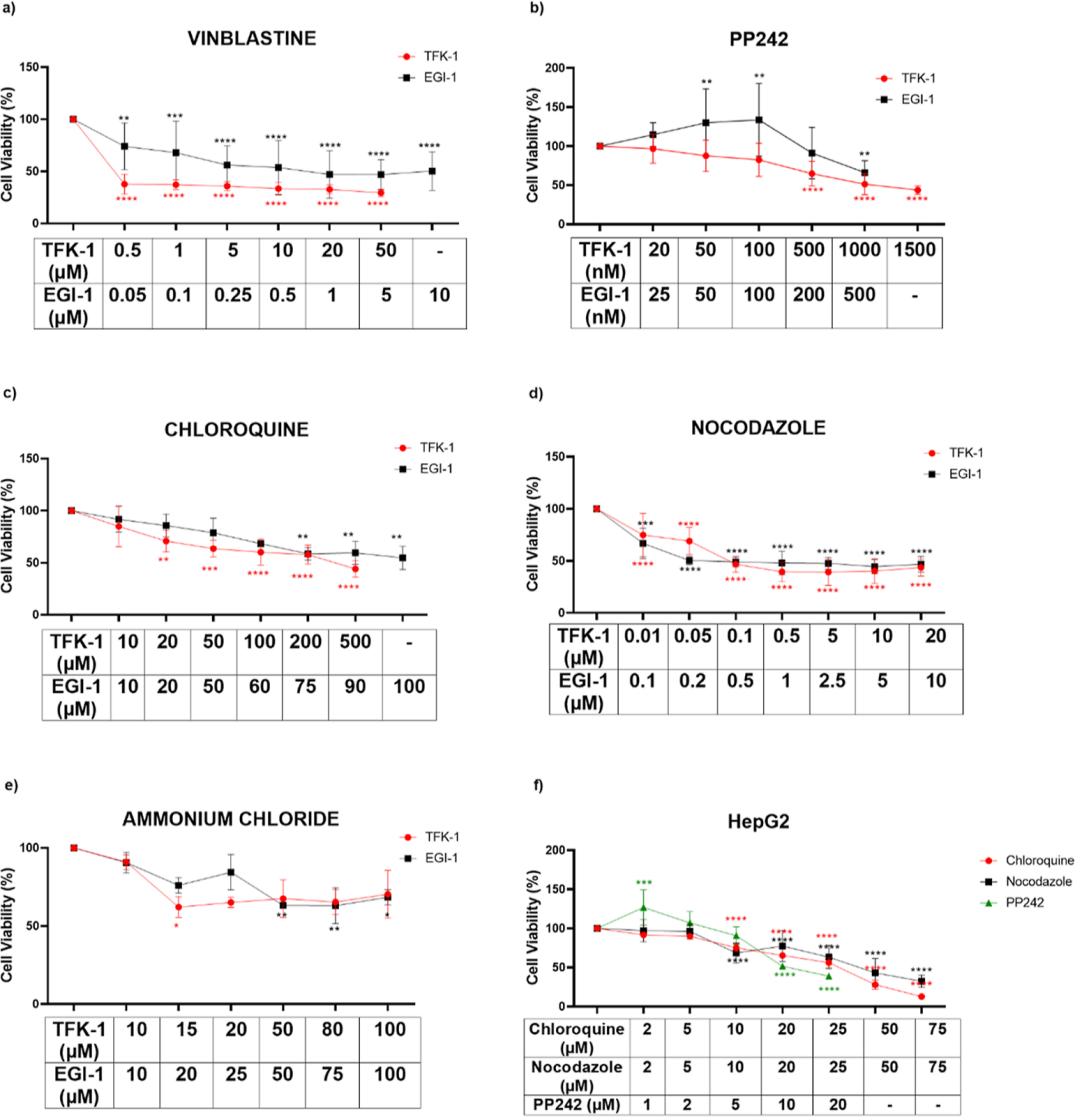
Cytotoxicity effect of autophagy modulators on TFK-1, EGI-1, and HepG2 cells. (a) Vinblastine, (b) PP242, (c) chloroquine, (d) nocodazole, (e) ammonium chloride treatment on the proliferation of TFK-1 and EGI-1 cells, and (f) chloroquine, nocodazole, and PP242 treatment on the proliferation of HepG2 cell. Each set of experiments was averaged and statistical analysis was performed using two-way ANOVA by the Dunnett’s test. These results represent data from samples in triplicate across three independent experiments (*n* = 3). *P < 0.05, **P < 0.01, ***P < 0.001 and ****P < 0.0001.

Previous studies demonstrated that the decrease in HDAC activity in cancer cells is related to the expression of autophagy regulators.^11,24^ To explore whether such crosstalk exists in the CCA context, we sought to determine the growth inhibitory effects of a panel of autophagy modulators on TFK-1 and EGI-1. First, we checked the cytotoxicity of autophagy modulators, such as vinblastine, nocodazole, chloroquine, PP242, and ammonium chloride (Figure 2a–e). While nocodazole decreased the viability of the cells by 50% at 0.1 μM, chloroquine concentration higher than 100 μM decreased the viability by 50% in TFK-1 and EGI-1 cells (Figure 2c,d). The ammonium chloride and vinblastine did not significantly reduce the proliferation of both cell lines (Figure 2a–2e). However, the concentration of PP242 higher than 1000 nM inhibited the proliferation of the cells by 50% (Figure 2b). Our results demonstrated that among the modulators, all the autophagy inhibitors, vinblastine, nocodazole, ammonium chloride, and chloroquine inhibited the viability of CCA cells; however, we selected the most effective inhibitors as nocodazole and chloroquine. PP242 is an autophagy activator through inhibiting mTOR, which plays an active role in promoting tumor growth. This drug also decreased the cell viability of both cell lines. Then, we tested the selected autophagy modulators on the HepG2 cell line and then observed that chloroquine and PP242 decreased the viability of the cells by 50% with 5.71 μM while nocodazole decreased viability at 6.7 μM by 50% in HepG2 cells (Figure 2f). The IC30 values of chloroquine, nocodazole, and PP242 are calculated, as shown in Table 1. Out of the five autophagy modulators that were tested, the best three were selected as nocodazole, chloroquine, and PP242 for further experiments.

### In Vitro Combination of HDACis and Autophagy Modulators Induced an Antiproliferative Effect

**Figure 3:**
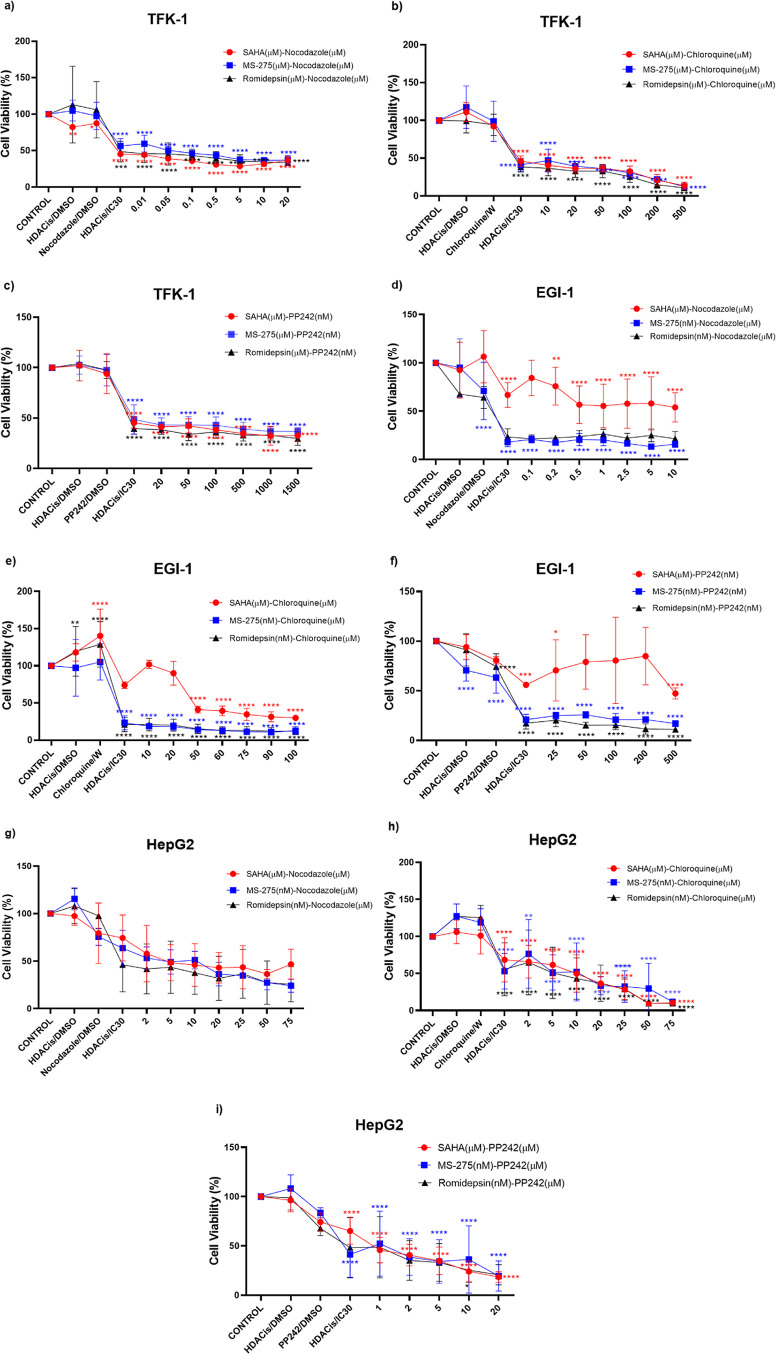

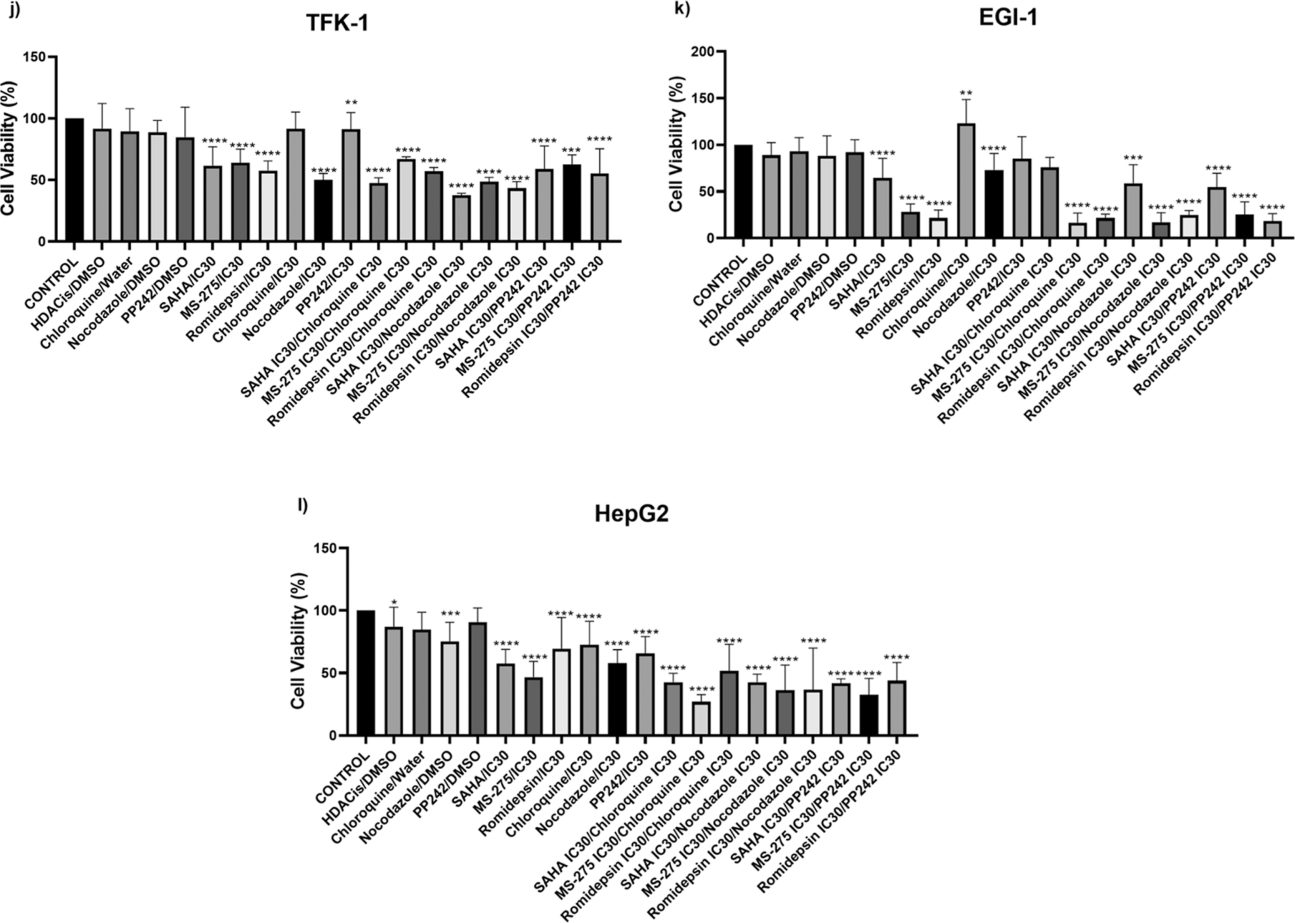
Antiproliferative effects of the IC30 of MS275, romidepsin, SAHA, each combined with increasing doses of nocodazole, chloroquine, and PP242 on TFK-1 (a–c), EGI-1 (d–f), and HepG2 (g–i) cells. IC30 combination of TFK-1 (j), EGI-1 (k), and HepG2 (l). These results represent data from samples in triplicate across three independent experiments (*n* = 3). *P < 0.05, **P < 0.01, ***P < 0.001 and ****P < 0.0001. W: Water.

To further understand the crosstalk between HDAC inhibition and autophagy, we determined the cytotoxic effect of HDACis and autophagy modulators by assessing the combinational treatment. First, we combined IC30 values of HDAC inhibitors with increasing concentrations of autophagy modulators to evaluate synergistic effects. The results showed that all combinations resulted in a decreased cell viability when compared to untreated controls and single HDACis treatment in TFK-1 (Figure 3a–c), EGI-1 (Figure 3d–f), and HepG2 cells (Figure 3g–i). The results from combinations of the IC30 values of HDACis and the autophagy modulators showed synergistic effects except for SAHA:nocodazole combination in the EGI-1. After determining the synergistic effect, as a second approach, the IC30 values of HDACis and the IC30 values of autophagy modulators were combined (Figure 3j–l). This approach was used to demonstrate the inhibition of cell growth by using only the IC30 values. This combination showed that the cell viability decreased prominently in nocodazole combinations compared with other combinations in all TFK-1, EGI-1, and HepG2 cell lines. For TFK-1, EGI-1, and HepG2 cells, SAHA:nocodazole, MS-275:nocodazole, romidepsin:nocodazole combinations decreased the cell proliferation of TFK-1 by 28, 12, and 13%; EGI-1 by 15, 11, and 1%; and HepG2 by 14, 11, and 20%, respectively. The results of this experiment showed that the combination of the HDACis IC30 with nocodazole IC30 demonstrated the best inhibitory effect on three cell lines. Therefore, in further experiments, we focused on the nocodazole:HDACis (MS-275, SAHA, and romidepsine) combinations.

### Combination of HDACis and Nocodazole Elicits Synergistic Antitumor Effects

The isobologram test was performed to investigate the combined synergistic effect of HDACis and nocodazole. The CI values for IC30 values of MS-275, romidepsin, and SAHA in combination with nocodazole are calculated and listed in Table 2 for TFK-1 and EGI-1 cells. The results revealed a synergistic cytotoxic effect for TFK-1 cells when IC30 of selected HDACis administered in combination with nocodazole lower than 20 μM. In EGI-1 cells, SAHA and nocodazole combinations revealed an antagonistic effect. However, the romidepsin:nocodazole and MS-275:nocodazole combination demonstrated a strong synergistic effect for EGI-1 cells.

**Table 2:** Combination Index (CI) Plots of TFK-1 and EGI-1 Cell Lines Treated with Combination Increased Doses of the Nocodazole with MS-275, SAHA, and Romidepsin^a^

	MS-275	SAHA	romidepsin	nocodazole	MS-275:nocodazole	SAHA:nocodazole	romidepsin:nocodazole
	dose (nM)	dose (μM)	dose (nM)	dose (μM)	CI value	CI value	CI value
TFK-1	3.5	2.25	3.7	0.01	0.15386	1.10215	0.00771
	3.5	2.25	3.7	0.05	0.09705	0.43653	0.02762
	3.5	2.25	3.7	0.1	0.08016	0.25157	0.03787
	3.5	2.25	3.7	0.5	0.25159	0.13268	0.08987
	3.5	2.25	3.7	5	0.73928	0.23162	0.43330
	3.5	2.25	3.7	10	1.24181	0.65930	0.68485
	3.5	2.25	3.7	20	2.29697	2.71316	1.14525
EGI-1	0.53	0.43	0.74	0.1	5.90 × 10^–4^	90,627.5	0.00643
	0.53	0.43	0.74	0.2	4.84 × 10^–4^	1854.71	0.00777
	0.53	0.43	0.74	0.5	8.76 × 10^–4^	2.29507	0.01205
	0.53	0.43	0.74	1	0.00380	2.04718	0.02172
	0.53	0.43	0.74	2.5	0.00166	8.70976	0.02664
	0.53	0.43	0.74	5	0.00217	17.1652	0.06347
	0.53	0.43	0.74	10	0.00510	17.9205	0.08374

a CI < 1—synergistic, CI = 1.0–1.1—additive, or CI > 1.1—antagonistic effects.

### Effect of Autophagy Inhibitor Combination with HDACis on Cell Cycle Progression

**Figure 4:**
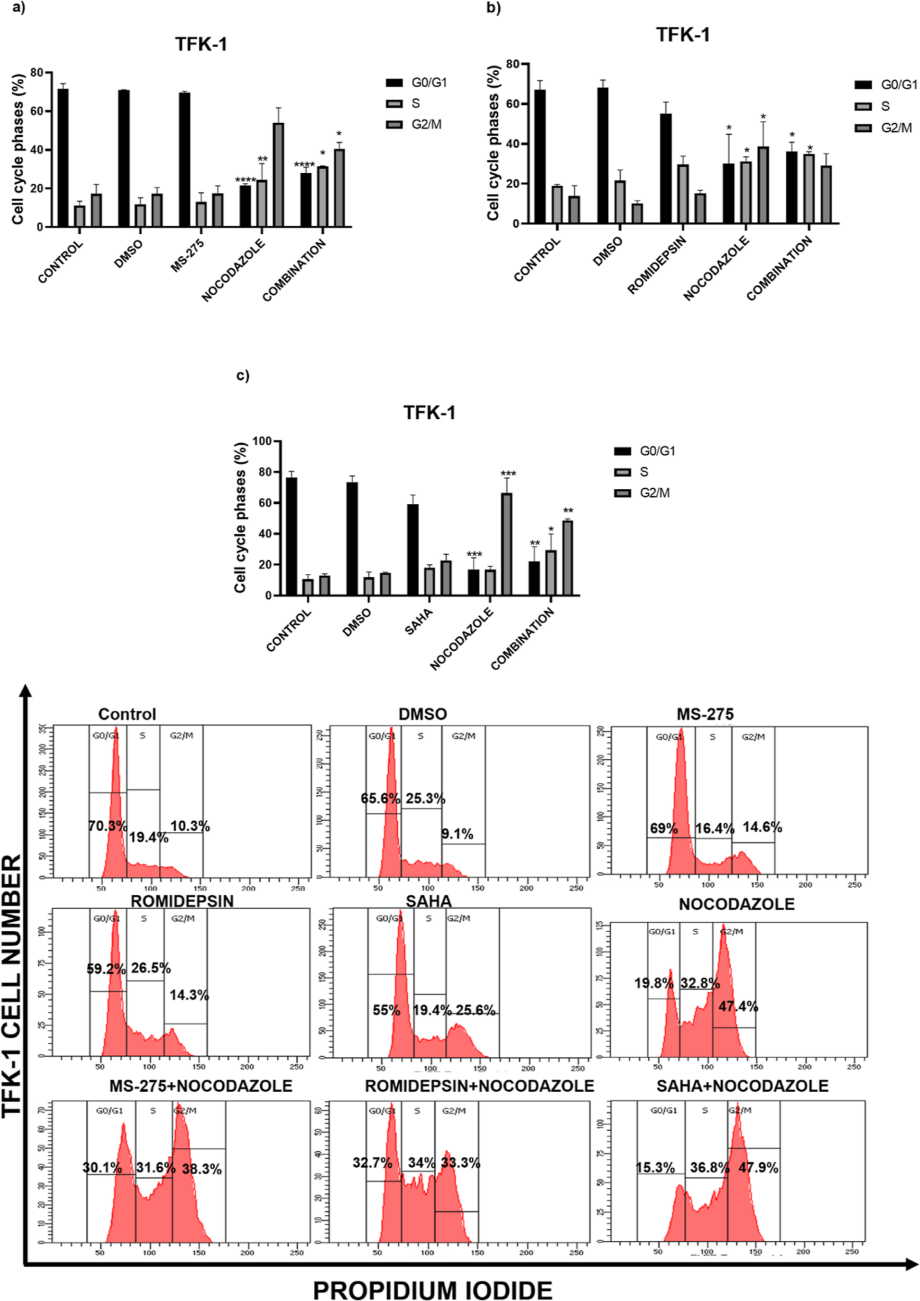

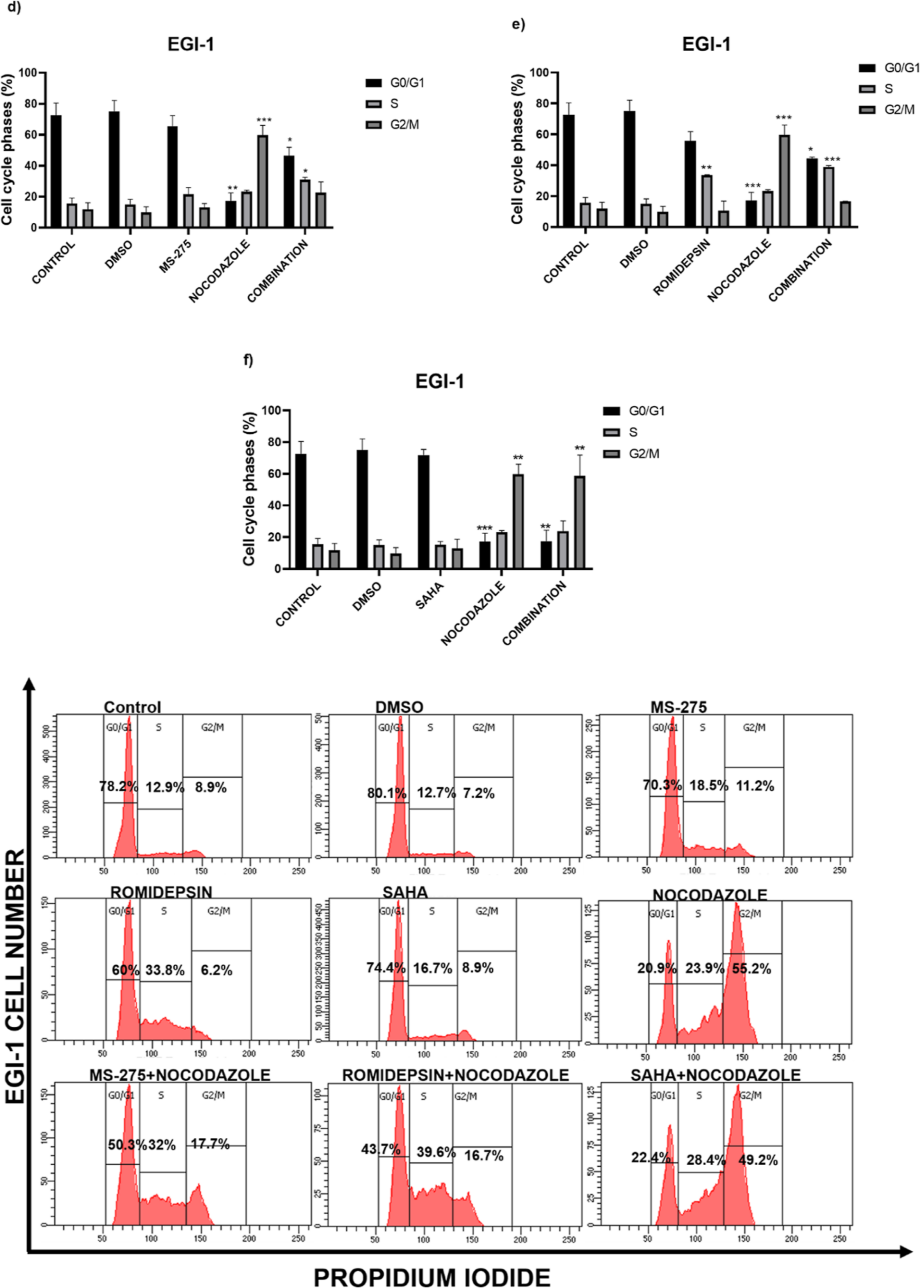
Effects of nocodazole with MS-275, romidepsin, and SAHA on cell cycle distribution in TFK-1 (a–c) and EGI-1 (d–f) cells. Histograms display the percentages of cell populations accumulated in each phase of the cell cycle. *P < 0.05, **P < 0.01, ***P < 0.001, and ****P < 0.0001 vs control. The data are represented by a mean percentage ± SE from replicates.

In order to evaluate the mechanism behind the growth inhibitory effects of combinational treatments, we investigated the impact of nocodazole and HDAC inhibitors on the cell cycle distribution of CCA cells. The results demonstrated that nocodazole treatment arrested the cells prominently at S and G2/M phases when compared to the control (Figure 4). When TFK-1 cells were treated with MS-275, no cell cycle arrest was observed (Figure 4a). When compared to single HDACis treatment, the TFK-1 cells were arrested at the S phase (10.6%) in response to romidepsin treatment (Figure 4b). In contrast, only SAHA administration induced the cells at S (7.6%) and G2/M (9.65%) phases (Figure 4c). To sum up, the nocodazole:HDACis (MS-275, SAHA, romidepsin) for TFK-1 cells caused the accumulation of cell population at the S phase (20.6, 18.8, and 20%, respectively) and G2/M phase (23.4, 35.6, and 15.2%, respectively). In EGI-1 cells, while single MS-275 caused a slight cell cycle arrest at the S phase compared to the control (Figure 4d), romidepsin treatment arrested the cells at the S phase (18.2%) (Figure 4e). Likewise, SAHA demonstrated no cell cycle arrest for EGI-1 cells (Figure 4f). However, the combination treatment of nocodazole:HDACis in EGI-1 cells caused an accumulation at the S (15.5, 8.4, and 23.6%, respectively) and G2/M phases (10.8, 46.8, and 4.8%, respectively) compared to untreated control cells. These results demonstrated that the combinations of HDACis with the autophagy inhibitor, nocodazole, arrested the CCA cells in S and G2/M phases. Consequently, nocodazole and romidepsin combination induced a more prominent cell cycle arrest than other combinations and thus, these drugs were used in further experiments.

### Nocodazole in Combination with Romidepsin and MS-275 Promotes Apoptosis in CCA Cell Lines

**Figure 5:**
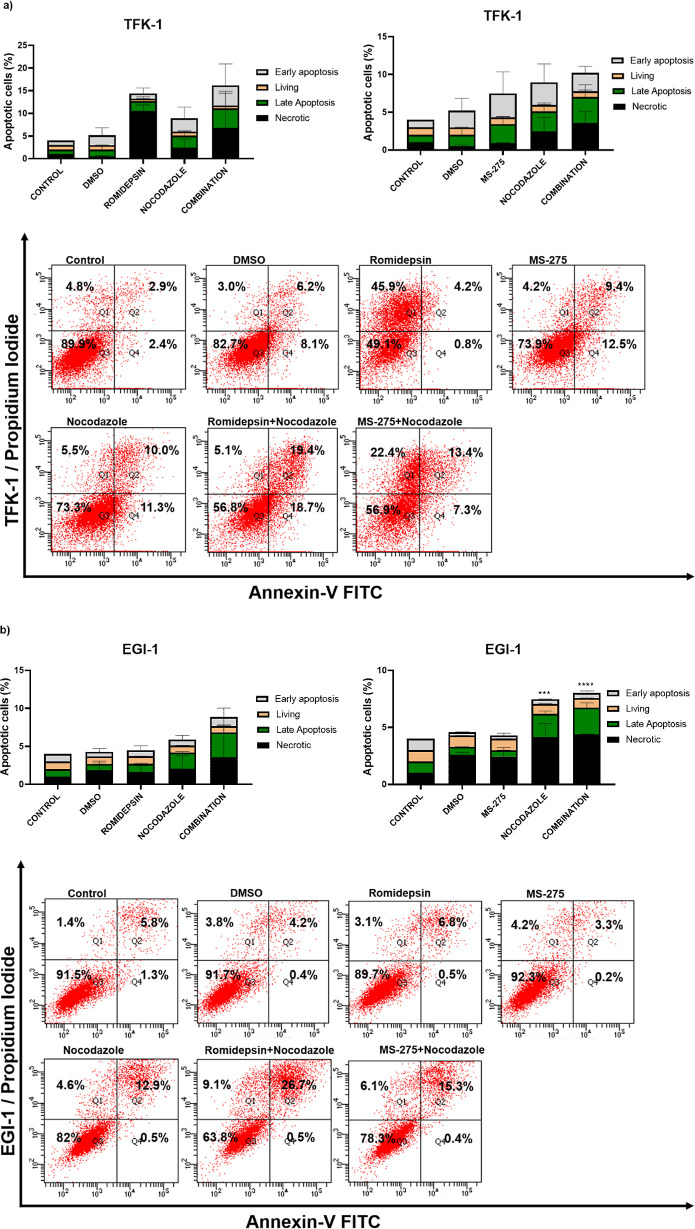
Nocodazole combined with romidepsin and MS-275 promotes apoptosis in TFK-1 (a) and EGI-1 cells (b). In the histograms, the cells in the lower right (Q4; Annexin V-FITC^+^/PI^–^) and upper right (Q2; Annexin V-FITC^+^/PI^+^) quadrants indicate early and late apoptosis, respectively. The lower left and the upper left quadrants show the living (Q3; Annexin V-FITC^–^/PI^–^) and necrotic cells (Q1; Annexin V-FITC^–^/PI^+^), respectively. ***P < 0.001 and ****P < 0.0001 vs control. The data are represented by a mean percentage ± SE from replicates.

We further wanted to investigate whether apoptotic cell death is involved in decreased cell viability. For this purpose, we measured the apoptotic cell death after treating the cells with the combination of romidepsin and MS-275 with nocodazole. The percentage of early and late apoptotic cells increased in combination treatments as compared to the control in TFK-1 and EGI-1 cells (Figure 5). The total apoptotic cell population of TFK-1 cells was increased in response to romidepsine:nocodazole combination by 16.8 and 33.1%, compared to single nocodazole and single romidepsin treatments, respectively. Romidepsin treatment only caused an increase in the necrotic cell population by 41.1% compared to the control (Figure 5a). In the other combination experiment where MS-275 and nocodazole were used, the total apoptotic cell population in single treatments of MS-275 and nocodazole was increased by 16.6 and 16%, respectively. However, the combination treatment also increased the necrotic cell population approximately 4-fold compared to the control and single treatments (Figure 5a). On the other hand, the results of combination treatment for EGI-1 cells demonstrated that the total apoptotic cell population in the romidepsine:nocodazole combination was increased by 13.8 and 19.9% respectively, compared to single nocodazole and romidepsin treatment (Figure 5b). The apoptotic and necrotic populations in a single treatment of MS-275 did not change significantly compared to the control. However, in combination, necrotic and apoptotic cell populations increased approximately 5-fold and 3-fold, respectively (Figure 5b). Romidepsin treatment only revealed similar results with TFK-1 cells in terms of the necrotic cell population. To our surprise, the MS-275:nocodazole combination did not seem to induce apoptosis significantly compared to single nocodazole. The apoptosis assessment showed that the combination of HDACis with nocodazole mostly increased the total apoptotic and necrotic population in CCA cells.

### The Combination of HDACis with Nocodazole Altered Protein Acetylation

**Figure 6:**
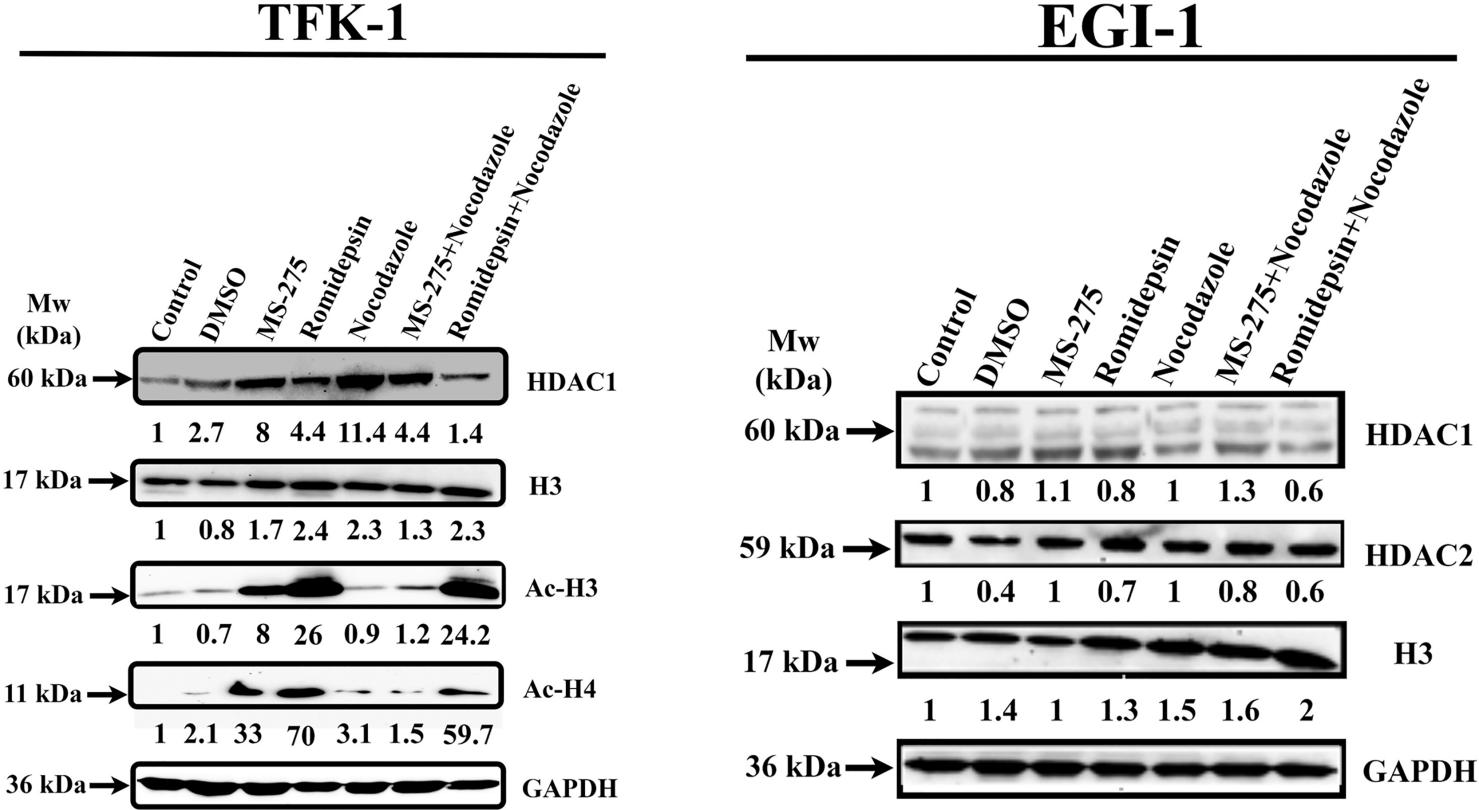
Differential effects of romidepsin, MS-275, and combination of nocodazole on acetylation H3 and H4, total H3 and HDAC1/2 in TFK-1, and EGI-1 cells after 48 h of treatment. GAPDH was used as a loading control.

To understand the effect of HDAC inhibition and nocodazole treatment on protein acetylation levels, western blotting was performed following drug treatment to observe the changes in protein expression levels of HDAC1, H3/Ac-H3, and Ac-H4. The results revealed that only MS-275 and romidepsin treatmentsignificantly increased Ac-H3 and Ac-H4 levels in TFK-1 cells (Figure 6). Moreover, the combination of romidepsin:nocodazole, but not MS-275:nocodazole treatment, induced histone 3 acetylation. MS-275 and nocodazole increased HDAC1 levels 6-fold and 14-fold compared to the control. In addition to these, in MS-275:nocodazole combination, the HDAC1 level was reduced compared to the single nocodazole. Also, the AcH3/H4 levels in this combination were significantly reduced compared to single MS-275. In the romidepsin:nocodazole combination, HDAC1 levels decreased compared to the single treatments, and the AcH3 level did not change compared to romidepsin only. However, the Ac-H4 level was significantly reduced compared to the romidepsin. Contrary to these, in the EGI-1 cells, HDAC1/2 did not change significantly compared to the control. However, especially the H3 protein expression level in the romidepsin and nocodazole combination demonstrated a 2-fold increase when compared to the untreated control (Figure 6).

## Discussion

In recent years, the modifications of histone proteins, which form the basic structure of the nucleosome, have been demonstrated to have a role in the control of biological processes, such as aging and development. Although it has been shown that many genes are silenced with promoter methylation in the hepatocarcinogenesis process, the role of histone code changes is not yet known.^25,26^ HDACis inhibit histone deacetylase enzymes and cause the accumulation of acetyl groups in histone proteins. These enzymes become defective by changing cellular processes in cancerous cells and high acetylation levels are observed that inhibits tumors.^27,28^ In this study, we investigated the effects of the combination of HDAC inhibitors and autophagy modulators on TFK-1 and EGI-1 CCA cell lines.

For the treatment of CCA cell lines, different classes of HDAC inhibitors, such as MS-275, romidepsin, SAHA, and PCI-34051 were utilized. Among these inhibitors, SAHA, which was used as a control in our study, was approved by the FDA for the treatment of cutaneous T-cell lymphoma patients. SAHA is known to inhibit the activity of all 11 HDACs classified as class I–II HDACs. Some findings have demonstrated that single SAHA or 5-fluorouracil (5-FU) and cisplatin combination inhibited cell proliferation for various cancer types, such as larynx,^29^ lung,^30^ breast,^31^ and different cholangiocarcinoma cell lines.^14^ In line with previous studies, our study showed that SAHA inhibited the proliferation of TFK-1 and EGI-1 CCA cells. Li et al. showed that romidepsin, which is another FDA-approved inhibitor, reduced the proliferation of TFK-1, EGI-1 cells, and different CCA cell lines.^32^ Our results revealed similar findings in the literature. Another synthetic HDAC inhibitor, MS-275, potently inhibits histone deacetylases in several human tumor cells, which supports our findings that show reduced cell proliferation in TFK-1 and EGI-1 cells treated with MS-275 alone and in combinations.^33^ In TFK-1 and EGI-1 cells, the selective HDAC8 inhibitor, PCI-34051^34^ showed a significant reduction at 10 μM in both cell lines. Consistent with this study, our results also demonstrated that MS-275 and romidepsin showed the best effect at low micro and nanomolar levels. Besides, in our previous study, tubastatin A, which is an HDAC6 inhibitor showed to reduction in cell growth by 50% at 15 and 20 μM concentrations for TFK-1 and EGI-1 cells, respectively.^35^

Cancer cells have evolved to adapt to themselves to survive by autophagy, which is a multistage death mechanism. It has been reported that autophagy modulators used in CCA cells promote cell death.^36,37^ The autophagy modulators such as chloroquine and ammonium chloride for autophagosomal degradation, nocodazole and vinblastine for autophagosome–lysosome fusion, and PP242 for the mTOR inhibitor were utilized in this study. In our study, the combinations of the HDACis with increasing doses of autophagy modulators showed that the best combinatorial effect with nocodazole combinations. According to the isobologram analysis, we propose that using a combination of a high concentration of nocodazole with SAHA is not recommended due to the antagonistic effect on EGI-1 and TFK-1 cells. However, the combination of a low concentration of nocodazole with MS-275 and romidepsin has a synergistic effect on both cell lines. These findings are consistent with the study showing that the combination of HDACis and autophagy modulators provide a synergistic effect on the breast cancer.^38,39^ Nocodazole, which is a prototypic microtubule inhibitor, has been shown to suppress the G2/M phase. Yamanaka et al. and Chi et al. have demonstrated that this inhibitor shows a similar effect on the lung and different cholangiocarcinoma cell lines in line with our findings.^40,41^

In this study, when the EGI-1 cells were treated with MS-275 at varying doses between 0.1 and 1 μM, the cells accumulated significantly in the G0/G1 phase, and an increase in the cell population was observed in the G2/M phase. In the literature, the accumulation of TFK-1 cells in the G0/G1 phase under the same conditions was demonstrated but more prominent suppression at the G2/M phase was shown compared to EGI-1 in response to MS-275.^33^ This could be due to the different concentration administration because Bardari et al. administered a higher concentration- and time-dependent treatment of MS-275 and hence, revealed more significant results. However, in our study, MS-275 slightly arrested EGI-1 cells in S and G2/M phases. The similar treatment in our study did not demonstrate any change for TFK-1 cells. In MS-275 and nocodazole combination, on the other hand, the accumulation in S and G2/M phases was suppressed in both cell lines. When the TFK-1 and EGI-1 results were compared, we observed different results that could be seen between cell lines despite belonging to same type of cancer. This could be probably due to the genetic differences in these cell lines. It was observed that the percentage of suppression in the G2/M phase increased with increasing concentrations of romidepsin (0–20 nM) on different CCA cell lines.^32^ In our study, the accumulation of TFK-1 cells was observed in S and G2/M phases with 3.7 nM of romidepsin. Besides, for EGI-1 cells, a slight increase in S and G2/M phases was observed when 0.74 nM of romidepsin was administered. These results were supported by the finding of an increased percentage of the G2/M phase in CCA cells in response to increasing concentration of romidepsin treatment.^32^ The combination of romidepsin:nocodazole induced an increase in S and G2/M phases compared to single romidepsin treatment. The results obtained in response to SAHA treatment revealed a suppression at S and G2/M phases for both cell lines; however, in the EGI-1, the arrest at the G2/M phase was more prominent. Studies in line with the results of our study show that SAHA is suppressed in the G2/M phases in lung, prostate, and breast cancer types.^42,43^

Previous studies show that only romidepsin treatment leads to a dose- and time-dependent induction of total apoptosis and necrosis.^44−46^ Romidepsin increased necrotic population levels in TFK-1 cells in the current study. Contrary to the findings for TFK-1 cells, romidepsin did not lead to an increase in the necrotic population on the EGI-1. Nocodazole was shown to induce apoptotic cell death in CCA cell lines similarly in different solid cancer types, like lung cancer.^47^ Du et al. showed that MS-275 treatment was shown to induce dose-dependent apoptosis in malignant ascites cells.^48^ The clinical or in vitro studies further demonstrate MS-275 as a potent time and dose-dependent growth inhibitor and cytotoxic agent against human tumor cells. However, in our study, MS-275 did not cause remarkable apoptosis induction. In literature studies, we can argue that the induction of apoptosis increases in line with dose and time dependencies. Contrary to this, romidepsin in the pharmacodynamic study has increased apoptosis but did not correlate with histone H3 acetylation levels.^27^

To assess the activities of HDAC enzymes and the acetylation level of histones, western blotting was performed. Treatment of tumor cells with romidepsin did not reduce HDAC1 and HDAC2 activities.^49^ Another study is contrary to this finding and demonstrates a significant decrease in HDAC1/2 activity after romidepsin treatment.^50^ In our study, treatment of tumor cells with romidepsin and MS-275 did not reduce HDAC1 activities, especially in TFK-1 cells, whereas HDAC2 activity was reduced in EGI-1 cells. Similar to our data, the research shows an increase in acetylated-H3 and -H4 by romidepsin and MS-275.^51,52^ On the other hand, despite our expectations, which was an increase in the acetylation level of histone proteins with the combination of nocodazole, our results did not show an increase except for the combination with MS-275. In addition, studies in the literature show that there are genetic and epigenetic alterations among CCA cell lines, including TFK-1 and EGI-1 cells that are used in the current study.^53^ Such genetic and epigenetic differences among the cell lines that in turn cause different gene regulation might be the reason for differential HDAC protein expression levels in TFK-1 and EGI-1 cells.

In conclusion, the limited treatment options in CCA show that investigating new approaches is necessary. In summary, combinations of different HDAC inhibitors and autophagy modulators have been studied in this study. Our results showed that HDACis and autophagy modulators have proliferation–inhibitory effects on CCA cell lines. This study creates a novel and unique approach for targeting CCA with a synergistic effect that will emerge with the combination of autophagy and HDAC inhibitors.

## Notes

The data sets generated during and/or analyzed during the current study are available from the corresponding author on reasonable request.
